# A pilot study of the impact of an integrated individual- and family therapy model for self-harming adolescents on overall healthcare consumption

**DOI:** 10.1186/s12888-021-03375-z

**Published:** 2021-07-26

**Authors:** Moa Bråthén Wijana, Inna Feldman, Richard Ssegonja, Pia Enebrink, Ata Ghaderi

**Affiliations:** 1 Department of Clinical Neuroscience, Karolinska Institutet, Stockholm, Sweden; 2 Department of Neuroscience, Child and Adolescent Psychiatry, Uppsala, Sweden; 3 Department of Public Health and Caring Science, Uppsala University, Uppsala, Sweden; 4 Department of Epidemiology and Global Health, Umeå University, Umeå, Sweden; 5 Department of Medical Sciences, Respiratory, Allergy- and Sleep Medicine Research Unit, Uppsala University, Uppsala, Sweden

**Keywords:** Adolescents, Self-harm, Healthcare consumption, Cost of treatment

## Abstract

**Background:**

Self-harming behaviors in adolescents cause great suffering and can lead to considerable costs to the healthcare system. The aim of the current study was to investigate the cost of an integrated individual and family therapy (Intensive Contextual Treatment: ICT) and to compare the adolescent’s healthcare consumption 1 year before and 1 year after treatment.

**Method:**

The study had a within group design with repeated measures. The clinical outcomes and the cost of ICT treatment are based on a sample of 49 participants who were previously enrolled in an intervention trial. Participants with significantly improved clinical outcomes (self-harm behavior, or general mental health symptoms) were defined as treatment responders. Calculation of changes in healthcare consumption is based on 25 participants who gave their consent to participate in a retrospective collection of healthcare data from medical records, including inpatient and outpatient care, and prescribed medication.

**Results:**

The average estimated cost of ICT per person was €5293. There were no significant differences between the cost of healthcare consumption 1 year before and after ICT, but the results suggested that the adolescents consumed less inpatient and specialized care after treatment. There was a significantly higher cost of psychotropic medication after treatment explained by a higher consumption of central stimulants. Treatment responders (general mental health problems) reduced their consumption of healthcare resources significantly more than non-responders, especially regarding hospital visits and total health care costs.

**Conclusions:**

Good response to the ICT in terms of improved general mental health symptoms seems to be associated with reduced healthcare consumption during the post-treatment period. However, controlled studies with larger sample sizes are needed to draw causal conclusions. The results of this study should be interpreted with caution as it is based on a small sample and attrition rate was high.

**Trial registration:**

This study has been registered with the ISRCTN: 15885573.

## Introduction

Self -harm is an important public health concern. It is a common and potentially life-threatening behavior [1, 2]. The term self-harm is not easily defined, given its varying types, motives, and meaning for different individuals, as well as different contexts in which it occurs [3, 4]. In this paper, the broader definition used in the guideline form the National Institute for Health and Care Excellence (NICE) will be used: “Self-poisoning or self-injury, irrespective of the apparent purpose of the act”. A meta-analysis of community-based studies from 41 countries between 1990 and 2015 showed that the overall lifetime prevalence of self-harm was 16.9% among adolescents [5]. In addition to negative mental and physical impact, self-harm imposes considerable financial burden for the healthcare sector as well as for the society in general. Costs can be either direct healthcare costs such as inpatient and outpatient care, psychopharmacology, psychotherapy and management of wounds, or indirect costs in terms of the consequences of morbidity, such as absence from school or impairment in social life [2, 6, 7]. Furthermore, self-harm in terms of self-poisoning and self-injury are common reasons for emergency ward visits, especially for young people. Moreover, self-harm behaviors tend to be repeated and increase the risk of completed suicide [8]. There have been some attempts to estimate societal costs of self-harm but the lack of systematic collection of data is aggravating. In the UK for example, an estimation from a large register of people admitted to general hospital as a consequence of self-harm, showed that the average healthcare cost for each episode was about £809 [9]. Another study showed that the overall healthcare costs per patient were significantly higher in a six-months period around an episode of self-harm [2]. Furthermore, the healthcare costs increased exponentially in relation to increasing episodes of self-harm and the expenses were mainly attributed to inpatient psychiatric care and psychotropic medication [2].

There is some evidence that self-harm tends to decline in late adolescents and young adulthood [10]. However, a recent population-based longitudinal study found that self-harm in adolescence was associated with increased prevalence of social disadvantage, anxiety and substance abuse in later life. This observation suggests that interventions addressing multiple risk domains should be considered when helping self-harming adolescents to adjust to adult life [10]. A recent longitudinal Swedish study also showed that adolescents reporting repetitive non-suicidal self-injury (≥5 episodes) are at substantial higher risk of negative outcomes such as stress and anxiety and that they report a lower life satisfaction in young adulthood [11]. Moreover, adolescents presenting with suicidality in addition to self-harm constitute a particularly vulnerable group as they consume even more care and have higher odds of most mental disorders and of being prescribed psychotropic medication [12].

There are surprisingly few trials for adolescents with self-harm, given the severity of the problem in minors [13, 14]. Randomized controlled trials have shown that some treatments have the potential of reducing self-harm in adolescents. However only Dialectical Behavior Therapy for adolescents (DBT-A) has been independently replicated [14, 15]. Currently, few treatments have been adapted to meet the needs of adolescents with complex psychosocial problems. Intensive contextual therapy, ICT [16] was developed in Uppsala county council in collaboration with the local policymakers and clinicians, where the main purpose was to fill the gap between in- and outpatient treatment and prevent residential care and long-term hospitalization. Preliminary results indicate that ICT reduces the need for inpatient and institutional care [17]. The adolescents also reported a significant reduction of self-harm, internalizing- and externalizing behavior symptoms, and a rise in general functioning in terms of school adjustment [17].

The target group for ICT is adolescents with complex psychosocial problems, where previous outpatient care failed to produce favorable outcome. Sometimes the only remaining options for this group are expensive inpatient or institutional care. In Sweden, the costs of institutional care for adolescents have increased for a long time, partly due to the increase of unaccompanied minors. Sweden has more than 2000 homes for care and residence for children and young people. Residential care homes specialized at treatment are essentially privately run and managed [18]. According to various sources, the estimated cost per day in residential care homes is €470–570 and the average lengths of stay is 60–70 days. This means that the average cost of a placement is about €28,000 [19, 20]. During 2018, just over 11 per 10,000 inhabitants, of the 11–17 year-olds were in need of psychiatric inpatient care and the average cost of a day in inpatient care in Sweden was about €1300 [21].

Taken together, the literature suggests it is important to establish whether brief interventions with intensified use of resources can lead to a reduced need for costly healthcare resources such as hospitalization in the longer term.

The aim of the present study was to report the costs of the ICT and the differences in healthcare consumption of patients during 1 year before and 1 year after treatment, in relation to the overall treatment effects. We hypothesized that the total costs of healthcare consumption will decrease during the year after completed treatment compared to the year before, and that treatment responders will reduce their healthcare consumption significantly more than the non-responders.

## Method

### Trial design

The study is based on data collected from the ICT-trial, ethics application (Dnr 2011/1593–31/5), with additional application approved by the Regional Ethical Review Board in Stockholm (Dnr: 2018/1902–32). The study was conducted in line with research ethics based on Declaration of Helsinki [22] regarding human experimentation and Swedish Research Council ethical principles. More detailed information on the study design, procedure, and outcomes has been presented elsewhere [17]. Health outcomes were collected at baseline, post treatment, as well as 6- and 12-month follow-ups. Data on ICT treatment costs were routinely collected during the trial for all 49 study participants. Data on healthcare consumption were collected retrospectively, during one-year pre-treatment, during the treatment period and during one-year post-treatment, from medical records of the 25 study participants who had provided informed consent for the current study.

### Study sample

In total, 49 participants were recruited via child and adolescent psychiatry in Uppsala county between January 2012 and July 2016. Inclusion criteria were: aged 13–19, repetitive self-harm behavior within the past 3 months, defined as both deliberate self-poisoning and self-injury, or suicidal thoughts, threats or plans. The adolescents also had to live together with at least one primary caregiver. Exclusion criteria were: reported severe psychiatric disorder (e.g., schizophrenia) requiring intensive in-patient stabilization (as assessed at baseline with a semi-structured diagnostic interview), insufficient comprehension of the Swedish language, severe substance abuse (to an extent that affects cognitive capacity and ability to generalize skills), or developmental disabilities.

**Fig. 1:**
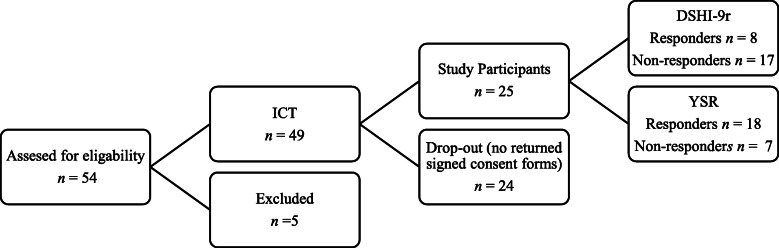
Flow-chart of the study sample construction

From the pool of 49 original participants, four declined participation, and two could not be reached. Of the 43 remaining, 18 persons were willing to participate (oral consent) but did not return the written informed consent, despite repeated reminders. We ended up with 25 participants who provided written consent to medical records access for investigating the impact of treatment on healthcare consumption (see Fig. 1).

### Intervention

ICT is a short-term (three to 6 months) intensive, and manualized psychiatric outpatient treatment. ICT is based on principles from Dialectical Behavior Therapy, DBT [23] and Functional Family Therapy, FFT [24] including youth, family and parent components. The four core targets of ICT are to increase: 1) the frequency of effective emotional regulation, 2) functional communication within the family, 3) school attendance or other scheduled activities, and 4) to devise a plan which clearly points to the maintenance of skills and action required in case of relapse. The targets are being obtained through intensive work with focus on active techniques such as roll-plays, chain-analysis and exposure, balanced with more supportive techniques like validation and reframing. Typically, FFT interventions used are defining and communicating hierarchical patterns and relational needs within the family as well as enactment, meta comments, and communication training. DBT interventions commonly used are skills training in emotion regulation, relations and mindfulness practice. Every case is assigned both a youth and a family therapist, the arrangements and constellations in treatment can therefore be adapted with the aim of achieving synergies. As mentioned earlier, one of the main purposes of ICT is to prevent residential care and inpatient admissions. Besides working with individual strategies like emotion regulation a great effort is made to strengthening the caregivers. Through a salutogenic approach (i.e. focus on the resources and abilities of the family), caregivers are encouraged to lower their expressed emotions (i.e., being less criticizing and more supportive and reduce overinvolvement). Family relations characterized by cohesion and trustful communication has proven to have a positive impact on adolescent’s well-being and functioning [25]. The parallel work with two therapists enables the families to feel secure and to acquire confidence for handling critical situations, without always having to seek emergency care. Yet another aspect of ICT is that it is an outreach treatment, conducted in the family’s home(s). This approach prevents drop-outs and promotes participation of all family members. The dose of treatment is a minimum of one family session and one individual session per week. For provider characteristics, see the section about intervention costs.

### Clinical outcomes

#### Measure of self-harm (primary outcome)

Improvement in self-harm behavior was measured by Deliberate Self-Harm Inventory, DSHI-9r, developed by Lundh and colleagues [26]. The nine items present common types of self-injuries such as cutting, burning or scratching the skin or banging the head. The respondent answers how many times the suggested behavior has occurred in the past 6 months, from one to more than five (scored as six), thus the maximum score is 54. The instrument has shown good test-retest reliability [27]. The internal consistency in the present sample was good (α = .82) To be able to detect changes during the treatment period the time frame was changed in the present study, from the past 6 months to the past month.

#### Measure of general mental health problems (secondary outcome)

Improvement in general mental health problems was assessed by Youth Self-Report, YSR [28]. This is a 112 item self-rating scale, for adolescents 11–17 years, to assess different behavioral problems. The 112 statements are scored on a three-point Likert-scale ranging from not true = 0, sometimes true = 1, and often true = 2. The questionnaire provides scores for seven different diagnostic and statistical manual of mental disorders (DSM-IV) oriented scales, which in turn can be categorized into one of two different subscales, internalizing behaviors or externalizing behaviors. The psychometric properties of YSR have proven to be good, with a test-retest reliability of the total scale of .87 and an internal consistency of .95 [29]. Cronbach’s alpha in the present study for YSR, total scale was .70. Also, for YSR the time frame was changed from the past 6 months to the past month. The primary outcome was the DSHI-9r- and the secondary outcome was the YSR.

Reliable change index, RCI, was calculated to estimate if the magnitude of change produced by the treatment was clinically significant. As proposed by Jacobson and Truax, the differences between the pre- and posttreatment scores were divided by the standard error of the differences between the two scores. If the value is greater than 1.96 it is considered as indicating improvement and if it also passes the clinical cut-off score it is considered as indicating recovery [30]. In this study we used clinical outcomes at 12- months follow-up. The treatment outcomes in the present study were the proportion of participants who scored as “improved or recovered” at 12-months follow-up and were considered as responders to treatment for the DSHI-9r, and YSR measures respectively.

### Costs

All the unit costs were collected in Swedish Krona and adjusted to Euro 2019 prices using purchasing power parities and inflation indices [31]. The costs are limited to direct healthcare costs. Indirect/societal costs as for example parental use of sick child leave was not registered systematically and therefore any estimates from the collected data were deemed highly uncertain.

### Intervention costs

The ICT treatment costs were calculated as opportunity costs, with the assumption that the time the therapists spent on conducting ICT, could otherwise have been spent on regular outpatient practice [32]. During the trial, the data on time spent for every session and treatment provider for every study participant, including travel time were routinely collected. The occupational groups that performed ICT were family therapists certified in FFT with a bachelor’s degree, nurses specialized in psychiatry, a psychologist, a psychiatric aide and counselors. The ICT-team participated in a consultation once every second week and had 30-min appointments with the case manager, three times for each patient during the whole treatment period. The time for the consultation team attendance were calculated for the total period of the study, which was 40.5 months. Normally, consultation teams are paused during vacation and national holidays for approximately 3 months, hence 9 months is used as effective time. Two consultation sessions per month, each 2 h long multiplied by 40.5 months gives a total of 162 h consultation per therapist during the whole study period. The cost of these hours was then added to the total costs of the treatment for all study participants. The costs for case management were calculated for each patient by multiplying one and a half hour with the hourly wages for the three participating professionals.

The costs for transportation included fuel consumption and private leasing of the cars, including tax and insurance, and was calculated as the average cost per kilometer. All unit costs used in the calculation are presented in Table 1.

**Table 1:** Values of the unit cost for each medical and non-medical service

	Unit costs (Euro, 2019)	Source
**ICT treatment**		Statistics Sweden
Psychologist (per hour)	48	as above
Specialist nurse (per hour)	45	as above
Counselor (per hour)	34	as above
Psychiatric aide (per hour)	33	as above
Case Manager (per hour)	48	as above
Consultation (per hour)	158	Uppsala Academic Hospital
Rental car (per hour)	3	Uppsala Academic Hospital
Fuel (per 100 km)	19	Swedish Tax Agency
**Health care consumption**		
Admission (per DRG)	4155–4886	Swedish Association of Local Authorities and Regions
Medical doctor at the hospital (per visit)	383	Swedish Association of Local Authorities and Regions
General practitioner (per visit)	274	as above
Psychologist (per visit)	326	as above
Specialist nurse (per visit)	211	as above
Other medical specialist (per visit)	221	as above
**Prescribed medication**	Various	Dental and Pharmaceutical Benefits Agency

*DRG* diagnosis related group

No set-up costs were calculated, since the study was conducted within the framework of regular treatment.

### Healthcare consumption

#### Inpatient and outpatient care

Data on healthcare resource use were collected from clinical records of the 25 study participants. Inpatient care was classified as psychiatry and other, outpatient care was divided into psychiatry and other hospital care (visits to doctors, psychologists and others) and primary care (visits to doctors and others). Inpatient care costs were estimated based on the specific reasons for admissions according to Diagnosis Related Group (DRG) codes and yearly DRG weights, which were provided by the Swedish National Board of Health and Welfare. Each DRG weight was assigned an average unit cost value on a yearly basis, provided by the Swedish Association of Local Authorities and Regions [33] for the period 2011–2016. DRG cost estimate = DRG weight x average monetary value per DRG unit. Outpatient care costs were estimated using national Swedish tariffs provided by Swedish Association of Local Authorities and Regions [33]. In order to calculate healthcare costs, the frequencies of resources/services units used were multiplied by their corresponding unit cost (i.e., the cost of a certain medical treatment). Unit costs and sources are presented in Table 1.

#### Prescribed medication costs

Prescribed medication costs were collected using patient medical records. Information about the Anatomical Therapeutic Classification (ATC) codes were used to calculate the price. Also, the amount and date of dispensed medication and dosages were collected. The medications were further classified based on ATC codes into psychostimulants (N06B), other-psychotropic (N05-N07, excluding N06B) and non-psychotropic (all other codes). Information about unit cost for each dispensed medication was obtained from the Swedish Prescribed Drug register [34].

### Statistical analyses

Multiple imputations were used to complete missing data for clinical outcomes at 12-months follow-up. The results for the different cost and frequency variables (i.e., number of hospital admissions, outpatient hospital visits, primary care visits, prescribed medication) were estimated during 1 year before the treatment, during the treatment and 1 year after the treatment. The differences in the mean number of hospital admissions, outpatient visits in hospital and primary care, prescribed medication and related costs between the pre-treatment and post-treatment periods were assessed using paired t-test and Wilcoxon Signed Rank Test for all study participants and then Mann-Whitney U Test separately for “responders” in comparison with “non-responder” for respective clinical outcomes, DSHI-9r, and YSR. Similarly, the treatment costs were also compared between respective groups of study participants, “responders” and “non-responders”.

## Results

### Participants and clinical outcomes

Sociodemographic and clinical characteristics for the 25 adolescents who participated in the study and the 24 who dropped out, are presented in Table 2. We conducted t-tests and chi square test to determine if there were significant differences between the groups. The only statistically significant difference regarding demographic characteristics between the two groups was the proportion of parents who reported having a university education. At pre-treatment the majority (72%) of the study participants reported five or more episodes of self-harm during the past month and in the drop-out group the corresponding proportion was 62.5%. It was also a rather large proportion (44% among the study participants and 50% in the drop-out group) who reported previous suicide attempt(s). At 12 months follow-up, there were 32% treatment responders (8 of 25 participants) for DSHI-9r and 72% (18 of 25 participants) for YSR.

**Table 2:** Demographic characteristics for the study participants and the drop-out group. The figures are percentage unless noted otherwise

	Study participants (*n* = 25)	Drop-out (*n* = 24)	Sig		Study participants (*n* = 25)	Drop-out (*n* = 24)	Sig
Adolescents’ age: M (SD)	14.4(1.1)	14.8(1.4)	>.05	**Drugs for mental health problems**^a^	84%	58.3%	>.05
Female gender	92.0%	79.2%	>.05	**Psychiatric diagnosis**^a^	68%	37.5%	>.05
Living with mother & father	54%	50%	>.05	**Been victimized/traumatized**	60.0%	66.7%	>.05
Parents university education^a^	14.3%	52%	.001	**Previous suicide attempt**	44%	50%	>.05
Parents employed^a^	92.9%	95.6%	>.05	**Self-harms episodes ≥ 5**	72%	62.5%	>.05
Households’ gross income (EUR)^a^				**Combination SH and SA**	24%	37.5%	>.05
Reported by the mothers: M (SD)	3309(1386)	4880(1922)	>.05	**Clinical outcomes**			
Reported by the fathers: M (SD)	3881(1370)	5443(2133)	>.05	Treatment responders, DSHI-9r	32%	60%	>.05
Experiencing income sufficient^a^	54.2%	45.8%	>.05	Treatment responders, YSR	72%	30%	<.05

^a^reported by the parents, *SH* self-harm, *SA* suicide attempts, *DSHI-9r* deliberate self-harm inventory, *YSR* youth self report

### Intervention costs

Operating costs were calculated for staff time spent in sessions and travels, consultations, case management and transportation. The average cost per study participant was €5293, SD = €2031 (min = 3515, max = 12,307, Mdn = 4436). We have also calculated intervention costs for drop-outs, €5802, SD = €2515 (min = 2351 max = 10,479, Mdn = 4338) and the costs for participants and drop-outs were not significantly different U(N_study participants_ = 25, N_drop-out_ = 24) = 240, z = − 1.20 *p* > .05, see Table 3. The intervention costs were not significantly different between YSR- responder (€5277) vs non-responder (€5334), U(N_responders_ = 18, N_non-responders_ = 7) = 61, z = −.21, *p* > .05. However, the treatment costs were borderline significantly higher for DSHI-9r responder compared with non-responder €6826 vs €4572, U(N_responders_ = 8, N_non-responders_ = 17) = 101, z = 1.92 *p* = 0.057.

**Table 3:** Health care consumption and related costs (in Euro, 2019) and visits 1 year before, during, and 1 year after treatment

	Treatment		Before treatment		After treatment		Difference between before and after treatment			sig
	Mean (SD)	Median (min, max)	Mean (SD)	Median (min, max)	Mean (SD)	Median (min, max)	Mean (SE)	Median (min, max)	(Location shift 95% CI)	
Intervention costs, study participants, *n* = 25	5293 (2031)	4436 (3512; 12307)								>.05
Intervention costs, drop-outs, *n* = 24	5802 (2515)	4338 (2351; 10479)								
Intervention costs, responder YSR, *n* = 18	5277 (2128)	4526 (3515;8694)								>.05
Intervention costs, non-responder YSR, *n* = 7	5334 (1915)	4235 (3515;8694)								
Intervention costs, responder DSHI-9r, *n* = 8	6826 (2878)	6840 (3741;12307)								**.057**
Intervention costs, non-responder DSHI-9r, *n* = 17	4572 (916)	4235 (3515;6800)								
**Health care consumption, n = 25**										
**Hospital care**										
**Inpatient care**										
Psychiatry										
Visits	0.36 (0.64)	0 (0; 2)	0.32 (0.63)	0 (0; 2)	0.28 (0.54)	0 (0; 2)	−0.04 (0.16)	0 (−2; 2)	(−4.5e-5; 3.9e-6)	>.05
Costs	1865 (5046)	0 (0; 24,269)	1293 (2634)	0 (0; 8742)	1942 (5543)	0 (0; 27,162)	649 (1248)	0 (− 8071; 27162)	(− 3.5e-6; 1.4e-6)	>.05
Other medical specialities										
Visits	0.12 (0.33)	0 (0; 1)	0.32 (0.56)	0 (0; 2)	0.08 (0.28)	0 (0; 1)	−0.24 (0.13)	0 (−2; 1)	(− 2.1e-5; 8.3–5)	>.05
Costs	219 (607)	0 (0; 2046)	557 (1019)	0 (0; 3496)	177 (632)	0 (0; 2742)	−379 (256)	0 (− 3496; 2742)	(− 2.3e-6; 2.7e-6)	>.05
**Outpatient care**										
Psychiatry										
Doctor										
Visits	1.48 (1.33)	1 (0; 5)	3.16 (2.87)	2 (0; 11)	2.84 (3.12)	0 (0; 11)	−0.32 (2.95)	0 (−5; 8)	(−1; 2)	>.05
Costs	555 (498)	383 (0; 1915)	1164 (1026)	766 (0; 3619)	1000 (1087)	766 (0; 3925)	− 164 (207)	0 (− 1869; 2776)	(− 383; 766)	>.05
Psychologist										
Visits	1.00 (1.68)	0 (0; 6)	5.60 (7.26)	3 (0; 28)	8.32 (14.53)	0 (0; 57)	2.72 (2.89)	−2 (−25; 48)	(− 1; 3)	>.05
Costs	272 (438)	0 (0; 1632)	1620 (2140)	707 (0; 7797)	1656 (3009)	0 (0; 13,560)	36 (625)	− 380 (− 6819; 10624)	(−7.8e-6; 761)	>.05
Other professionals e.g. psychiatric nurses										
Visits	6.48 (17.96)	2 (0; 91)	15.00 (22.50)	5 (0; 105)	13.28 (19.07)	7 (0; 68)	−1.72 (5.51)	0 (−97; 63)	(−5; 5)	>.05
Costs	959 (2653)	211 (0; 13,374)	2173 (3090)	740 (0; 12,860)	2043 (2866)	932 (0; 9159)	−130 (778)	0 (−11,528; 8450)	(−682; 709)	>.05
Other medical specialities										
Doctor										
Visits	0.96 (1.02)	1 (0; 3)	2.32 (2.87)	1 (0; 9)	1.40 (1.66)	1 (0; 7)	−0.92 (0.58)	0 (−8; 6)	(−3.8e-5; 1)	>.05
Costs	368 (391)	383 (0; 1149)	849 (1058)	383 (0; 3448)	413 (418)	383 (0; 1533)	−436 (197)	0 (− 3065; 766)	(−4.3e-6; 383)	>.05
Other professionals e.g. nurses										
Visits	0.88 (2.74)	0 (0; 13)	1.40 (2.55)	0 (0; 9)	2.32 (4.13)	0 (0; 18)	0.92 (0.71)	0 (−6; 13)	(−1.0; 1.8e-5)	>.05
Costs	156 (541)	0 (0; 2665)	238 (511)	0 (0; 1989)	404 (857)	0 (0; 3968)	166 (140)	0 (− 1326; 2884)	(−46; 4.7e-6)	>.05
**Total hospital visits**	11.28 (19.35)	7 (0; 100)	28.12 (27.58)	20 (0; 119)	28.52 (27.50)	15 (0; 88)	0.40 (6.86)	−2 (−104; 61)	(−10; 11)	>.05
**Total hospital costs**	4395 (6368)	2051 (0; 28841)	7895 (6632)	6416 (0; 25684)	7632 (9789)	5667 (0; 48340)	−263 (2211)	− 1128 (− 18,900; 39798)	(− 1961; 4590)	>.05
**Primary care**										
Doctor										
Visits	1.88(2.47)	1 (0; 11)	1.92 (2.69)	0 (0; 10)	2.24 (2.33)	1 (0; 8)	0.32 (0.70)	0 (−10; 7)	(−1.0; 4.3e-5)	>.05
Costs	448 (622)	273 (0; 3011)	364 (483)	0 (0; 1490)	469 (463)	274 (0; 1662)	105 (133)	0 (− 1337; 1662)	(−274; 1.1e-06)	>.05
Other professionals e.g. nurses and psychologists										
Visits	3.32(4.33)	2 (0; 21)	3.04 (4.09)	1 (0; 16)	4.04 (3.75)	3 (0; 12)	1.00 (0.82)	1 (−12; 9)	(−3.0; 5.0e-5)	>.05
Costs	524 (619)	432 (0; 2322)	461 (733)	211 (0; 3099)	603 (630)	474 (0; 2169)	142 (165)	183 (− 2416; 1728)	(− 474; 11)	>.05
**Total primary care visits**	5.20 (5.40)	4 (0; 24)	4.96 (6.37)	3 (0; 26)	6.28 (4.88)	6 (0; 19)	1.32 (1.43)	1 (−22; 16)	(−5; 1)	>.05
**Total primary care costs**	973 (926)	777 (0; 3905)	821 (1053)	484 (0; 4436)	1074 (905)	1102 (0; 3831)	253 (284)	244 (− 3754; 3391)	(−840; 69)	>.05
**Total visits**	16.48 (23.33)	10 (1; 124)	33.08 (29.90)	25 (2; 129)	34.80 (26.99)	22 (4; 89)	1.72 (7.05)	0 (−107; 59)	(−15; 10)	>.05
**Total health care costs**	5367 (6504)	2704 (383; 29,546)	8716 (6947)	7681 (484; 26941)	8705 (9684)	7342 (809; 48340)	− 11 (2211)	− 1977 (− 19,010; 39283)	(−2653; 4450)	>.05
**Medication consumption (costs),** ***n*** **= 25**										
Psychiatry			231 (265)	172 (0; 1058)	518 (431)	410 (0; 1488)	287 (98)	274 (− 699; 1462)	(− 481; −30)	**<.05**
Central stimulants			120 (199)	0 (0; 744)	301 (382)	0 (0; 1266)	181 (78)	0 (− 525; 1266)	(− 314; 6.2e-5)	>.05
Other psychiatric drugs e.g. antipsychotics			111 (131)	68 (0; 533)	217 (339)	93 (0; 1488)	106 (69)	2 (− 174; 1462)	(−93; 26)	>.05
Other non-psychiatric drugs e.g. antibiotics			49 (77)	15 (0; 335)	63 (76)	31 (0; 240)	14 (21)	13 (− 311; 240)	(−31; 7)	>.05
**Total medication costs**			284 (274)	213 (0; 1058)	579 (442)	454 (0; 1577)	305 (103)	253 (− 604; 1509)	(−474; −47)	**<.01**

*YSR* youth self report, *DSHI-9r* deliberate self-harm inventory, sig significance, *SD* standard deviation, *SE* standard error, *CI* confidence interrval

### Healthcare consumption

The healthcare resources consumed during the three assessment periods (one-year pre-treatment, during treatment, and one-year post-treatment) are reported in Table 3. Treatment utilization of most hospital services declined during the post-treatment period, although none of those changes were statistically significant (Table 3). The overall reduction in inpatient admissions was the most visible finding. In comparison to pre-treatment, the number of doctors’ visits in psychiatry and other hospital-based care was also reduced during the post-treatment period. Contrary to that, the meetings with psychologists became more frequent after the treatment. Further, the consumption of primary care increased during post-treatment period, from 5.0 to 6.3 visits per person. Study participants used a notable amount of healthcare resources during the one-year pre-treatment period, €7895 per person for hospital care and €821 per person for primary care. Hospital based care costs decreased slightly during the post-treatment period while primary care costs increased, from €821 to €1074.

### Medication consumption

The mean expenditure of prescribed medication significantly increased during the post-treatment period, from €284 to €579 per person, mostly because of the increased consumption of central stimulant medications. During the post-treatment period, the study participants consumed more central stimulant medications with a total cost of €181 (see Table 3).

### Healthcare and medication consumption in relation to response to treatment

Comparison of healthcare and medication consumption between DSHI-9r–treatment responder vs non-responders showed no significant differences between the pre- and post-treatment periods (in contrast, costs for DSHI-responders were higher during the treatment period). The YSR-treatment responders reduced their consumption of healthcare resources significantly more than the non-responders (Table 4). The responders had significantly fewer outpatient visits to doctors and psychologists at the hospital, with 1.19 and 2.81 per person respectively. The number of hospital visits was reduced by 12.13 visits per person for responders, while it increased with 22.67 for the non-responders. The reduction in total healthcare costs amounted to €4,547 per responder, while the total healthcare costs increased by €8053 per non-responder. Contrary to that, there were no differences in changes in medication consumption between the pre- and post-treatment periods for YSR-responder vs non-responder.

**Table 4:** Changes in health care consumption and related costs (in Euro, 2019) between pre-treatment (1 year before) and post-treatment (1 year after) in relation to treatments effects

	Changes in health care consumption		Difference	
	Non-responders YSR	Responders YSR		sig
	Mean difference (SE)	Mean difference (SE)	M(SE)	
	*n* = 7	n = 18		
**Health care consumption**				
**Hospital care**				
Inpatient care				
Psychiatry	0.33(0.29)	− 0.25(0.17)	− 0.58(0.31)	>.05
Other	0.22(0.15)	− 0.50(0.16)	−0.72(0.24)	<.01
Outpatient care visits				
Psychiatry				
Doctor	1.22(1.08)	−1.19(0.62)	−2.41(1.15)	<.05
Psychologist	12.56(6.18)	−2.81(1.95)	−15.37(5.26)	<.01
Other	8.78(6.85)	−7.63(7.44)	−16.40(11.21)	>.05
Other				
Doctor	−0.78(0.76)	−1.00(0.82)	− 0.22(1.24)	>.05
Other	0.33(0.62)	1.25(1.07)	0.92(1.51)	>.05
**Total hospital visits**	22.67(7.80)	−12.13(8.40)	−34.79(12.68)	**<.05**
**Total hospital costs**	7789(4295)	− 4842(1895)	− 12631(4000)	**<.05**
**Primary care**				
Doctor	0.44(0.71)	0.25(1.04)	−0.19(1.49)	>.05
Other	1.33(1.12)	0.81(1.14)	−0.52(1.74)	>.05
**Total primary care visits**	1.78(1.56)	1.06(2.09)	−0.72(3.04)	>.05
**Total primary care costs**	221(295)	263(295)	42(598)	>.05
**Total health care costs**	8053(4211)	−4547(1895)	−12600(4000)	**<.01**
Medication consumption (costs)				
**Psychiatry**	400(168)	221(116)	− 179(200)	>.05
Central stimulant	147(98)	200(116)	53(168)	>.05
Other	253(158)	21(56)	−232(137)	>.05
**Other**	33(25)	13(31)	−20(45)	>.05
**Total medication costs**	432(168)	232(137)	−200(211)	>.05

*YSR *youth self report

## Discussion

### Main findings

The present study provides some insights and serves as a foundation for more informed and well-powered future studies. Our results suggest that patients who according to YSR responded to the ICT treatment reduced their healthcare consumption, especially the specialized care at the hospital. That ICT compares well with other treatments and can be a favorable option in terms of treatment cost is also an important factor for decision-maker to consider.

The management of self-harm occurs within a complex system of health and social care. Notably, ethical constraints make it difficult to conduct RCT-studies and hence compare costs and effects for new treatment options compared with usual care. The few studies that have looked at changes in healthcare consumption before and after different treatment programs summarized that outpatient efforts that are more intensive than usual care have promising prospects of paying off in the longer term [35]. Mainly, these effects are related to reduced inpatient care during the follow-up period [35].

When interpreting the results of this pilot study, it is important to bear in mind that they are based on a small sample, and that the attrition rate was high. The study can therefore be considered as underpowered. However, it fills an important gap of knowledge as cost evaluations of interventions for mental health conditions are scarce in general, and for self-harm in adolescents in particular.

### Costs of ICT

Costs of ICT treatment were estimated as €5293 per patient. The cost of Dialectical Behavior Therapy for adolescents (DBT-A), and enhanced usual care (EUC) was investigated by Haga and colleagues [36]. The treatments extended over approximately the equivalent time as ICT (19 weeks), but were associated with higher total treatment costs; €16,199 and €13,217, respectively [36]. In another study by Ougrin and colleagues [37], the mean total cost for supported discharge service (SDS), and usual care (UC) was £64,355 (€72,721), and £63,463 (€69,809) per patient respectively. However, both SDS and UC treatments lasted for 6 months [37] compared to the present study with a mean treatment time of 4.5 months. In a study with similar methodology as the present one, societal cost-of-illness was calculated 1 year before, during and after DBT for adults [35]. The direct healthcare cost associated with 1 year of DBT was €10,524, which is comparable to the costs for the ICT.

Within the ICT, the costs per patient varied substantially during treatment. Similar variations have also been found in the aforementioned studies. One reasonable explanation pertaining to the present study is that participants with more severe symptomatology required more frequent treatment appointments/visits. Another explanation is that the accessibility of patients and families varies. ICT is a flexible method where salutogenesis is important [16], hence the patients and their parents are encouraged to maintain daily activities whenever possible. In turn some families might not be able to schedule appointments with the same intensity as others.

Nearly half of the healthcare cost during the treatment period is driven by healthcare consumption besides ICT. This is in line with findings from other studies [37, 38] and also anticipated since adolescents with a high psychiatric symptoms load would not be expected to immediately change their consumption pattern of healthcare. Especially in the startup phase it is of great value to offer parallel services, until the patient has confidence in the ICT, and the same is probably true for the termination phase.

### Comparison of healthcare costs 1 year before and 1 year after ICT

We found no statistically significant differences between healthcare consumption 1 year before ICT and 1 year after ICT. The costs before and after treatment are remarkably similar, but when studying the figures in more detail, some trends emerge. The most important finding which also is in line with the aim of ICT, is a reduction of inpatient treatment, both psychiatric and other. In contrast to what has been observed in studies with adult patients, the proportion of patients receiving inpatient treatment is relatively small in the present study. Psychiatric hospitalization of adolescents has however a much higher threshold in Sweden and other countries as it is regarded as a drastic measure. A study with more patients and/or longer follow-up might have resulted in more distinct results.

Patients consumed more primary care resources, as well as more care provided by psychologists than by physicians and psychiatrists during the one-year follow-up. Lack of a control group makes it difficult to interpret the outcomes with certainty, but ICT has a pronounced aim to make the patients more willing to maintain a care plan, and to rely less on specialized care delivered as crisis interventions. From this point of view, a greater consumption of outpatient care such as psychological treatment or primary care can possibly be an attractive economic development in the long run.

The only statistically significant cost difference not considering responders vs non-responders, was medication consumption during pre- and post-treatment periods. The mean age in this sample was rather low and pharmacological treatment of adolescents sometimes might be associated with certain restrictions. It is therefore possible that the result reflects a diminished conservative attitude on the part of healthcare as the patients grow older. It is also important to note that a large part of the increased costs consists of central stimulants, CS, which are relatively expensive medications compared to for example selective serotonin reuptake inhibitors [34]. From an economic perspective though it might lead to savings in the long run if consumption of CS results in a higher level of functioning and reduction in mental health problems. A systematic review showed consistent evidence that pharmacotherapy for adolescents with ADHD were cost-effective compared to no treatment or behavioral therapy [39].

### Association between healthcare costs and treatment response

Among those who provided consent for the current study (*n* = 25), a greater proportion were treatment responders as measured by YSR than by DSHI-9r. It was also shown that the responders on DSHI-9r had significantly higher treatment costs than the non-responders. One intuitive explanation, is that a more intensive and consequently more expensive treatment has a higher potential when it comes to reducing self-harming behaviors, and also that a more frequently offered care signals to the patient that his/her situation is of concern. The patients might feel validated and attended to and in turn experience a reduced need to use self-harming behavior as a means to regulate emotion.

When comparing the costs between 1 year before and 1 year after ICT we didn’t detect any differences between DSHI-9r treatment responders and non-responders. It is however important to note that there was a greater proportion of responders on DSHI-9r in the drop-out group, and if included we might have seen other figures. On the other hand, treatment responders on the YSR consumed substantially less hospital care after ICT compared to the non-responders. YSR is a multidimensional scale and gives a broad picture of the symptomatology [28, 29], and as such it might be a better marker for the need of subsequent specialized care.

### Limitations

Several limitations need to be mentioned. First, we had no control group and thus we cannot draw firm conclusions about the effectiveness of ICT. Therefore, we cannot rule out that the data observed during and after ICT could also have been noted with non-specific care. However, given that the majority of the ICT participants had recurrent and repetitive self-harm in combination with suicidal behaviors and extensive internalizing and externalizing symptoms it is likely that they would have consumed an even larger amount of care without the ICT. Moreover, as mentioned in the introduction, a combination of self-harm and suicidality is associated with more severe outcomes [12]. Also, research suggests that self-harm reaches a peak around ages 15–16 [10]. The average age in this particular sample is rather low, which may imply that without treatment they might have developed an even more frequent self-harming behavior with subsequent serious consequences.

Second, the drop-out from this study by half of the participants has to be regarded as unfortunate and may limit the generalizability of the results. All the participants from the original study were introduced to the cost study by phone. The majority were willing and interested in participating, but they failed to return the signed informed consent forms. We have however no reason to suspect that the drop-out group is different in a significant way that would confound the conclusions as the base-line observed characteristics were essentially similar (see Table 2). We have reasons to believe that the high drop-out rate was mainly due to the formal, mandatory procedure in obtaining informed consent. The age group is not used to signing and returning written consents. A secure and more smooth electronic system for obtaining informed consent was being developed, but unfortunately, we could not wait for its implementation. Such a system makes it much easier for participants to provide consent and reduces the risk of drop-out due to the inconvenient procedure of posting consent forms. The statistical difference between the two groups (Table 2) regarding parental educational level contradicts what we would expect, namely that children of parents with a higher degree of education to a larger extent would value participating in a research study. We believe this difference is simply a random effect. Even if it cannot be classified as a baseline characteristic, the higher proportion of treatment responders (YSR) among study participants (Table 2) could potentially contribute to a distortion as those might have been more prone to consent”.

We have also limited the cost analyses to direct healthcare treatment costs and have not included other societal costs. Productivity losses due to parents’ staying at home from work, caring for their adolescents, or absenteeism from school could have given us a broader and most probably more positive picture of the cost differences before and after ICT.

Lastly, the patients in ICT may not be representative of the vast majority of adolescents with self-harm behavior due to their complex psychosocial symptoms hence why we cannot draw conclusions beyond this group.

### Clinical implications and future directions in research

Despite the mentioned limitations, this study includes register data and as such should be regarded as a less biased option when measuring healthcare consumption than self-reports. The study also has a strong ecological validity since it’s run in a specialized real-world treatment setting. The description of costs before, during and after ICT also fills an important knowledge gap, since we need a better understanding of the compositions of healthcare cost and how patterns of healthcare might change after treatment. This is in particular warranted when it comes to treatment that initially may impose a higher financial burden than usual outpatient care. Clinical outcomes are important and often the main focus of psychotherapy research, but in times when resources in healthcare are scarce in relation to needs it is important to be able to document healthcare utilization. As mentioned in the introduction, the estimated average cost of residential care homes in Sweden is €28,000 [19, 20]. We need a long-term follow-up period and a comparison group to ensure that the ICT-treatment may reduce needs for institutional care. In that case, every single avoided case of residential placement will pay for ICT-treatment for five patients.

The observed changes in healthcare consumption between treatment responders and non-responders also raise questions about what kind of measures can most accurately predict treatment success. A narrow focus on overt and observable behaviors such as self-harm might obscure our judgment and mislead our interventions.

For future research it would be of great value to have a larger sample size and use a longer follow-up, to be able to detect clearer patterns of healthcare consumption and conduct a full health economic evaluation. It would also be relevant to include societal costs such as costs due to non-completed education.

## Conclusions

The ICT treatment might have a potential to reduce healthcare consumption during the post-treatment period, especially for treatment responders with improvements in internalizing and externalizing symptoms and behaviors. Replications with a control condition, larger sample size, and longer follow-up period are needed.

## Data Availability

The datasets used and/or analyzed during the current study are available from the corresponding author on reasonable request.

## Abbreviations

ATC: anatomical therapeutic classification
DBT-A: dialectical behavior therapy for adolescents
DRG: diagnosis related group
DSHI: deliberate self-harm inventory
DSM: diagnostic and statistical manual of mental disorders
EUC: enhanced usual care
FFT: functional family therapy
ICT: intensive contextual therapy
RCI: reliable change index
RCT: randomized controlled trial
YSR: youth self-report

## References

[1] Nock MK (2010). Self-injury. Annu Rev Clin Psychol.

[2] Sinclair JM (2011). Healthcare and social services resource use and costs of self-harm patients. Soc Psychiatry Psychiatr Epidemiol.

[3] Edmondson AJ, Brennan CA, House AO (2016). Non-suicidal reasons for self-harm: a systematic review of self-reported accounts. J Affect Disord.

[4] Hawton K, Saunders KEA, O'Connor RC (2012). Self-harm and suicide in adolescents. Lancet.

[5] Gillies D, Christou MA, Dixon AC, Featherston OJ, Rapti I, Garcia-Anguita A, Villasis-Keever M, Reebye P, Christou E, al Kabir N, Christou PA (2018). Prevalence and characteristics of self-harm in adolescents: Meta-analyses of community-based studies 1990-2015. J Am Acad Child Adolesc Psychiatry.

[6] Bustamante Madsen L, Eddleston M, Schultz Hansen K, Konradsen F (2018). Quality assessment of economic evaluations of suicide and self-harm interventions. Crisis.

[7] Brent RJ (2008). Applied cost-benefit analysis.

[8] Grandclerc S, de Labrouhe D, Spodenkiewicz M, Lachal J, Moro MR (2016). Relations between nonsuicidal self-injury and suicidal behavior in adolescence: a systematic review. Plos One.

[9] Tsiachristas A, McDaid D, Casey D, Brand F, Leal J, Park AL, Geulayov G, Hawton K (2017). General hospital costs in England of medical and psychiatric care for patients who self-harm: a retrospective analysis. Lancet Psychiatry.

[10] Moran P, Coffey C, Romaniuk H, Olsson C, Borschmann R, Carlin JB, Patton GC (2012). The natural history of self-harm from adolescence to young adulthood: a population-based cohort study. Lancet.

[11] Daukantaite D, Lundh L-G, Wångby-Lundh M, Claréus B, Bjärehed J, Zhou Y, Liljedahl SI (2021). What happens to young adults who have engaged in self-injurious behavior as adolescents? A 10-year follow-up. Eur Child Adolesc Psychiatry..

[12] Bjureberg J, Ohlis A, Ljótsson B, D'Onofrio BM, Hedman-Lagerlöf E, Jokinen J, et al. Adolescent self-harm with and without suicidality: cross-sectional and longitudinal analyses of a Swedish regional register. J Child Psychol Psychiatry. 2019;60(3):295–304. 10.1111/jcpp.12967. 10.1111/jcpp.12967 PMC7379534 30207392

[13] Saunders KE, Smith KA (2016). Interventions to prevent self-harm: what does the evidence say?. Evid Based Mental Health.

[14] Ougrin D, Tranah T, Stahl D, Moran P, Asarnow JR (2015). Therapeutic interventions for suicide attempts and self-harm in adolescents: systematic review and meta-analysis. J Am Acad Child Adolesc Psychiatry.

[15] Asarnow JR, Mehlum L (2019). Practitioner review: treatment for suicidal and self-harming adolescents - advances in suicide prevention care. J Child Psychol Psychiatry.

[16] Nicholaisen L, et al. Intensiv kontektuell behandling av självskada (IKB): En integrerad individ- och familjebehandlingsmodell. 2010, Landstinget i Uppsala län och Uppsala kommun Uppsala.

[17] Wijana MB, Enebrink P, Liljedahl SI, Ghaderi A (2018). Preliminary evaluation of an intensive integrated individual and family therapy model for self-harming adolescents. BMC Psychiatry.

[18] Konkurrensverket (2017). Marknaden för hem för vård eller boende för ensamkommande och andra barn och unga.

[19] Socialstyrelsen, Individ- och familjeomsorg. Lägesrapport. 2019.

[20] Socialstyrelsen, Barn och ungas hälsa, vård och omsorg 2103. 2013: Västerås.

[21] landstig, S.k.o., Psykiatrin i siffror. Barn- och ungomspsykiatri- Kartläggning 2018. 2019: Stockholm.

[22] Association, W.M., World medical association Decleration of Helsinki (2001). Ethical priciples for medical research involving human subjects. Bull World Health Organ.

[23] Linehan MM (1993). Cognitive-behavioral treatment of borderline personality disorder.

[24] Alexander JF, Waldron HB, Robbins MS, Neeb AA (2013). Functional family therapy for adoelscent behavior problems.

[25] Halstead RO, Pavkov TW, Hecker LL, Seliner MM (2014). Family dynamics and self-injury behaviors: a correlation analysis. J Marital Fam Ther.

[26] Lundh L-G, Karim J, Quilisch E (2007). Deliberate self-harm in 15-year-old adolescents: a pilot study with a modified version of the deliberate self-harm inventory. Scand J Psychol.

[27] Bjarehed J, Lundh LG (2008). Deliberate self-harm in 14-year-old adolescents: how frequent is it, and how is it associated with psychopathology, relationship variables, and styles of emotional regulation?. Cogn Behav Ther.

[28] Achenbach TM (1991). Manual for youth self report.

[29] Gustle, L.H., Hansson K., Sundell K., Lundh L.G., Löfholm C.A., Blueprints in Sweden. Symptom load in Swedish adolescents in studies of functional family therapy (FFT), multisystemic therapy (MST) and multidimensional treatment Foster Care (MTFC). Nord J Psychiatry, 2007. 61(6): p. 443–451, doi: 10.1080/08039480701773196. 10.1080/08039480701773196 18236311

[30] Jacobson NS, Truax P (1991). Clinical significance: a statistical approach to defining meaningful change in psychotherapy research. J Consult Clin Psychol.

[31] CCEMG - EPPI - Center Cost Converter. 29-04-19; Available from: https://eppi.ioe.ac.uk/costconversion/default.aspx. [cited 2020 May 09]

[32] Drummond M, Sculpher MJ, Torrance GW, O'Brien BJ, Stoddart GL (2005). Methods for the economic evaluation of health care Programmes.

[33] Kostnad per patient, KPP. 2020. Available from: https://skr.se/ekonomijuridikstatistik/statistik/kostnadperpatientkpp.1076.html. Accessed 9 May 2020.

[34] Dental and Pharmaceutical Benefits Agency, TLV. 2020. Available from: https://www.tlv.se/in-english/medicines.html. Accessed 9 May 2020.

[35] Wagner T, Fydrich T, Stiglmayr C, Marschall P, Salize HJ, Renneberg B, Fleßa S, Roepke S (2014). Societal cost-of-illness in patients with borderline personality disorder one year before, during and after dialectical behavior therapy in routine outpatient care. Behav Res Ther.

[36] Haga E, Aas E, Grøholt B, Tørmoen AJ, Mehlum L (2018). Cost-effectiveness of dialectical behaviour therapy vs. enhanced usual care in the treatment of adolescents with self-harm. Child Adolesc Psychiatry Ment Health.

[37] Ougrin D, Corrigall R, Poole J, Zundel T, Sarhane M, Slater V, Stahl D, Reavey P, Byford S, Heslin M, Ivens J, Crommelin M, Abdulla Z, Hayes D, Middleton K, Nnadi B, Taylor E (2018). Comparison of effectiveness and cost-effectiveness of an intensive community supported discharge service versus treatment as usual for adolescents with psychiatric emergencies: a randomised controlled trial. Lancet Psychiatry.

[38] Mehlum L, Ramberg M, Tørmoen AJ, Haga E, Diep LM, Stanley BH, Miller AL, Sund AM, Grøholt B (2016). Dialectical behavior therapy compared with enhanced usual Care for Adolescents with Repeated Suicidal and Self-Harming Behavior: outcomes over a one-year follow-up. J Am Acad Child Adolesc Psychiatry.

[39] Wu EQ, Hodgkins P, Ben-Hamadi R, Setyawan J, Xie J, Sikirica V, du EX, Yan SY, Erder MH (2012). Cost effectiveness of pharmacotherapies for attention-deficit hyperactivity Disprder. A Systemtic literature review. CNS Drugs.

